# Double sequential external defibrillation versus standard defibrillation in refractory ventricular fibrillation: A systematic review and meta-analysis

**DOI:** 10.3389/fcvm.2022.1017935

**Published:** 2022-11-24

**Authors:** Yongkai Li, Xiaojing He, Zhuanyun Li, Dandan Li, Xin Yuan, Jianzhong Yang

**Affiliations:** ^1^Emergency Trauma Center, The First Affiliated Hospital of Xinjiang Medical University, Ürümqi, China; ^2^Seven Section of Department of Gynecology, The Second Hospital of Hebei Medical University, Shijiazhuang, Hebei, China; ^3^Department of Emergency Medicine, Union Hospital, Tongji Medical College, Huazhong University of Science and Technology, Wuhan, China

**Keywords:** cardiopulmonary resuscitation, double sequential external defibrillation, refractory ventricular fibrillation, prognosis, meta-analysis

## Abstract

**Introduction:**

Double sequential external defibrillation (DSED) in cardiopulmonary resuscitation has shown different results in comparison with standard defibrillation in the treatment of refractory ventricular fibrillation (RVF). This review aims to compare the advantages of DSED with standard defibrillation in the treatment of refractory ventricular fibrillation.

**Materials and methods:**

PubMed, Embase, Web of Science, and Cochrane Library were searched from inception to May 1, 2022. Studies included adult patients who developed RVF. The study used random-effects and fixed-effects models for meta-analysis, which was reported by risk ratio (RR) with 95% confidence interval (CI), mean difference (MD), or standardized mean difference (SMD). The risk of bias in individual studies was assessed using the Robins-I tool for observational studies and the Cochrane Risk of Bias 2 (ROB-2) tool for clinical trials. Primary outcomes included the termination of RVF, prehospital return of spontaneous circulation (ROSC), survival to hospital admission, survival to hospital discharge, and good neurological recovery. Secondary outcomes included age, total defibrillation attempts, emergency medical system arrival time, and dose of epinephrine and amiodarone used.

**Results:**

In this systematic review and meta-analysis, 10 studies containing 1347 patients with available data on treatment outcomes were included. The pooled estimate was (RR 1.03, 95% CI, 0.89 to 1.19; *Z* = 0.42, *P* = 0.678 > 0.05) for Termination of RVF, (RR 0.84, 95% CI, 0.63 to 1.11; *Z* = 1.23, *P* = 0.219 > 0.05) for ROSC, (RR 0.86, 95% CI, 0.69 to 1.06; *Z* = 1.4, *P* = 0.162 > 0.05) for survival to hospital admission, (RR 0.77, 95%CI, 0.52 to 1.15; *Z* = 1.26, *P* = 0.206 > 0.05) for survival to hospital discharge, (RR 0.65, 95%CI, 0.35 to 1.22; *Z* = 1.33, *P* = 0.184 > 0.05) for good neurologic recovery, (MD −1.01, 95%CI, −3.07 to 1.06; *Z* = 0.96, *P* = 0.34 > 0.05) for age, (MD 2.27, 95%CI, 1.80 to 2.73; *Z* = 9.50, *P* = 0.001 < 0.05) for total defibrillation attempts, (MD 1.10, 95%CI, −0.45 to 66; *Z* = 1.39, *P* = 0.16 > 0.05) for emergency medical system arrival time, (SMD 0.34, 95%CI, 0.17 to 0.50; *Z* = 4.04, *P* = 0.001 < 0.05) for epinephrine, and (SMD −0.30, 95%CI, −0.65 to −0.05; *Z* = 1.66, *P* = 0.1 > 0.05) for amiodarone.

**Conclusion:**

We discovered no differences between DSED and standard defibrillation in termination of RVF, prehospital return of spontaneous circulation, survival to hospital admission, survival to hospital discharge, good neurological outcome, emergency medical system arrival time, and amiodarone doses in patients with RVF. There were some differences in the number of defibrillations and epinephrine doses utilized during resuscitation.

**Systematic review registration:**

[https://www.crd.york.ac.uk/prospero/display_record.php?RecordID=329354], identifier [CRD42022329354].

## Introduction

Out-of-hospital cardiac arrest (OHCA) is the leading cause of death and disability in public health events worldwide (1, 2). Although excellent progress has been made in the field of cardiopulmonary resuscitation (CPR), the survival rate of patients treated with CPR remains low and many of those who survive have persistent neurological impairment. More than 350,000 adults die each year in the United States from out-of-hospital cardiac arrest, including 100,000 from ventricular fibrillation or pulseless ventricular tachycardia (VF/VT) (3). However, RVF is uncommon, poorly treated, and has a high mortality rate of 85% to 97% (1). RVF was defined as a patient presenting with ventricular fibrillation, having three consecutive standard defibrillation attempts, and remaining in ventricular fibrillation at the fourth rhythm analysis (4). DSED is a technique that uses two separate defibrillators and two sets of electrode pads, with the defibrillation pads placed in two different planes, usually anterolateral and anterior-posterior, to deliver two consecutive shocks in close proximity (5, 6). Current research in the treatment of RVF has shown promise for DSED in the management of RVF, in addition to conventional standard defibrillation. In recent years, there has been a growing number of studies comparing DSED with standard defibrillation, but the final results vary. Some studies have concluded that DSED has no benefit compared to standard defibrillation therapy for RVF in terms of survival, return of spontaneous circulation (ROSC), and neurological outcomes (7). However, some studies and case series suggest that DSED is superior to standard defibrillation in the prehospital return of spontaneous circulation and the termination of RVF. The early treatment of DSED may be associated with higher rates of refractory ventricular fibrillation termination and return of spontaneous circulation (4, 8).

To further deepen our understanding of the treatment and potential impact of DSED in patients with RVF, based on the knowledge of existing animal studies, case reports, and clinical research evidence, and to address the limitations of previous reviews, we conducted a systematic review and meta-analysis that focused on assessing two questions. (1) Primary outcome: Compared with standard defibrillation, is there an advantage in assessing double sequential external defibrillation on prehospital return of spontaneous circulation, termination of refractory ventricular fibrillation, survival to hospital admission, survival to hospital discharge, and good neurological recovery in patients with RVF? (2) Secondary outcomes: In addition to the observation of the primary outcome, age, number of defibrillations, emergency medical system arrival time, and dose of epinephrine and amiodarone used these measures have an impact on and improve the outcome? Neurological outcome was measured using the Glasgow–Pittsburgh cerebral performance category (CPC). CPC1∼2 were considered good neurological outcomes; CPC 3∼5 were defined as poor neurological outcomes (9, 10). Our goal was to build on published studies and hypothesize that these studies differ in quality, whether DSED is more advantageous in the treatment of RVF patients compared with standard defibrillation, and guide policy choices about how to choice of rescue methods and resources for RVF patients in the future.

## Methods

### Search strategy

We conducted and reported this systematic review and meta-analysis study protocol according to the criteria outlined in the PRISMA guidelines (11). The study protocol was registered with the PROSPERO International Prospective Systematic Evaluation Registry (registry number CRD42022329354; Centre for Reviews and Dissemination, University of York). We compared the use of DSED with standard defibrillation strategies, including randomized and non-randomized clinical trial designs and observational research studies.

The researcher (YL) searched four databases: PubMed, Embase, Web of Science, and Cochrane Library. There was no specific date, age, gender, or language restrictions. The coverage dates for this systematic review and meta-analysis start at the beginning of each database (PubMed, 1946; Embase, 1947; Web of Science, 1950 and Cochrane Library, 1995) and ends on May 1, 2022. Search criteria combined free text search exploded [Medical Subject Headings (MeSH) terms/EMTREE terms] and refractory ventricular fibrillation, Cardiopulmonary Resuscitation, Heart Arrest, Electric Countershock, and Double sequential external defibrillation. The search was initially developed for PubMed and then adapted to each of the other 4 databases by searching for medical subject term terms and mapping to other controlled vocabularies, ensuring that the search strategy was relevant and comprehensive.

### Study selection criteria

#### Exclusion criteria

(1) Consensus guidelines, animal studies, letters, case reports (<5 cases), or non-peer-reviewed or unpublished reports. (2) Patients whose families refused Cardiopulmonary Resuscitation (CPR); patients with cardiac arrest due to cachexia and trauma; patients with cardiac arrest due to drowning, pulseless ventricular tachycardia, hypothermia, and suspected drug overdose. (3) Literature with incomplete or inaccessible valid data.

#### Inclusion criteria

(1) Adult patients (≥18 years) in refractory ventricular fibrillation during cardiac arrest. (2) Patients eligible for RVF are defined as the initial presentation of VF and still in ventricular fibrillation at the time of analysis after 3 or more consecutive standard defibrillation attempts. (3) Literature published in English and available in the full article.

### Effect measures and synthesis methods

The literature was screened and managed using EndNote (version 20; Clarivate Analytics). Statistical methods were performed using Stata 14.0 (StataCorp LLC 4905 Lakeway Drive College Station, USA) software and RevMan 5.4 provided by the Cochrane Collaboration (RevMan 5.4; Cochrane Collaboration, Oxford, UK) software for meta-analysis. Differences in categorical outcomes were reported by risk ratio (RR) with 95% confidence interval (CI), mean difference, or standardized mean difference. The Q test (test level α = 0.1) combined with *I*^2^ was used to determine the magnitude of statistical heterogeneity in the literature. Meta-analysis was performed using a fixed-effects model when *P* ≥ 0.10 and *I^2^* < 50% were considered to have no statistical heterogeneity; Meta-analysis was performed using a random-effects model when *P* < 0.10 or *I^2^* ≥ 50% were considered to have statistical heterogeneity, and meta-analysis was performed after analyzing the source of heterogeneity and excluding the effect of significant clinical heterogeneity; when significant clinical heterogeneity existed, subgroup analysis or sensitivity analysis, or only descriptive analysis was performed.

### Data collection process and study risk of bias assessment

Two (YL and JY) investigators will independently evaluate each article and will perform title and abstract screening and full article screening, and in case of disagreement, resolve it through discussion or with the assistance of a third investigator. Information extracted included: author, year, research method, total sample size, gender, age, defibrillation mode, outcome events, survival to hospital discharge, and medication used. Tables were created using Microsoft Excel 2019 software to extract and record data from the literature. All studies considered eligible by title and abstract screening were reviewed in full by two independent reviewers (YL and JY) using the same criteria. The quality rating of each study was assessed independently by two reviewers (YL and JY), and any disagreements were subsequently resolved through discussion and involvement of a third reviewer (DL), and two (YL and JY) researchers independently assessed the risk of bias for the included studies. The risk of bias in individual studies was assessed using the Robins-I tool (12) for observational studies and the Cochrane Risk of Bias 2 (ROB-2) tool (13) for clinical trials.

## Results

### Study selection

**FIGURE 1:**
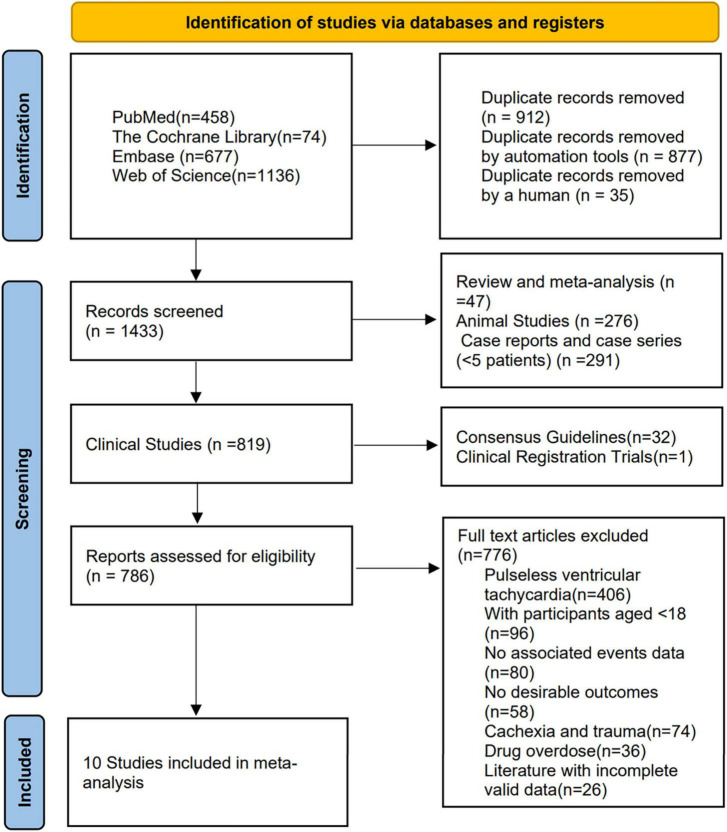
Flowchart of included studies.

A total of 2345 studies were found in the search of the four databases. After removing duplicates of 912 studies, 1433 studies were screened for eligibility. These included 276 studies from animal studies, 47 studies from reviews and meta-analyses, 291 studies from case reports and case series (<5 patients), 32 studies from consensus guidelines and reports, and one unpublished article from clinical registry trials. 786 studies were eligible for full-text review, 776 studies were finally excluded after reading the title and abstract content, and ten studies (4, 8, 14–21) met the inclusion criteria (Figure 1), with a total of 1347 patients included. Table 1 summarizes the study characteristics, design, and results.

**TABLE 1:** Characteristics of studies included in the meta-analysis.

Source	Research method	Groups	Cases	Gender (male/ female)	Age	Prehospital ROSC	RVF termination	Survival to hospital admission	Survival to hospital discharge	Good neurologic outcome	Defibrillation attempts	Dispatch to EMS arrival (minutes)	Epineph- rine (mg)	Amioda- rone (mg)
Emmerson et al. (17)	Observational analysis	Standard defibrillation	175	144/31	62.5 ± 16.5	61	NR	34	16	NR	10.4 ± 3.7	NR	NR	NR
		DSED	45	42/3	59.8 ± 13.8	17	NR	10	2	NR	13.6 ± 5.8	NR	NR	NR
Cheskes et al. (8)	Observational study	Standard defibrillation	201	170/31	63.8 (15.7)	43	157	NR	NR	NR	NR	NR	4.5 (2.1)	393.6 (80.1)
		DSED	51	43/8	61.8 (14.3)	9	39	NR	NR	NR	NR	NR	5.3 (1.9)	427.2 (54.5)
Merlin et al. (20)	Case Series	Standard defibrillation	NR	NR	NR	NR	NR	NR	NR	NR	NR	NR	NR	NR
		DSED	7	4/3	62(45-78)	NR	5	4	3	2	5.4(3-9)	NR	NR	NR
Cheskes et al. (4)	RCT	Standard defibrillation	36	28/8	64.4 (14.9)	9	24	NR	NR	NR	6.8(2.1)	NR	4.2 (2.0)	413.8 (65.3)
		DSED	55	49/6	64.4 (14.4)	22	42	NR	NR	NR	2.8 (2.2)	NR	4.1 (3.0)	385.7 (75.1)
Cabanas et al. (15)	Case Series	Standard defibrillation	NR	NR	NR	NR	NR	NR	NR	NR	NR	NR	NR	NR
		DSED	10	9/1	76.5(65–82)	3	7	0	NR	NR	NR	NR	NR	NR
Ross et al. (21)	Cohort analysis	Standard defibrillation	229	168/61	61.4	86	NR	81	33	26	NR	NR	NR	NR
		DSED	50	38/12	59.4	14	NR	16	4	3	NR	NR	NR	NR
Beck et al. (14)	Observational study	Standard defibrillation	239	174/65	62.3 ± 14.3	144	NR	117	49	NR	4.7 ± 1.9	NR	6.2 ± 3.9	NR
		DSED	71	61/10	62.2 ± 14.1	28	NR	25	10	NR	6.7 ± 2.3	NR	8.2 ± 4.2	NR
Mapp et al. (19)	Case–control study	Standard defibrillation	103	80/23	58.4 ± 13.3	42	NR	52	24	20	5 (4–5)	8 (6–10)	5 (4–6)	450 (450–450)
		DSED	25	22/3	58.3 ± 10.6	5	NR	12	4	3	7 (6–8.75)	8 (6–12)	6 (4.5–6.5)	450 (450–450)
Kim et al. (18)	Retrospective analysis	Standard defibrillation	21	17/4	65 (18–93)	NR	NR	6	3	7	7 (7–9.5)	7 (4–10)	8 (6–9)	450 (450–450)
		DSED	17	14/3	60 (18–83)	NR	NR	14	7	5	7 (6–10)	8.5 (6.8–11)	6 (2.5–10)	450 (375–450)
Cortez et al. (16)	Case Series	Standard defibrillation	NR	NR	NR	NR	NR	NR	NR	NR	NR	NR	NR	NR
		DSED	12	11/1	59	3	9		3	2	NR	NR	NR	NR

DSED, double sequential external defibrillation; IQR, interquartile range; SD, standard deviation; ROSC, return of spontaneous circulation; EMS, emergency medical service; RVF, refractory ventricular fibrillation; NR, not reported.

### Study characteristics

Table 1 summarizes the characteristics of all studies, the majority (9 of 10; 90%) were observational studies [four retrospective studies (8, 14, 17, 18), three case series (15, 16, 20), one cohort study (21), and one case-control study (19)], and only one randomized controlled trial (RCT) (4) met the final inclusion criteria, and all ten studies (4, 8, 14–21) were published in 2015 or later.

### Primary outcome

#### Return of spontaneous circulation

**FIGURE 2:**
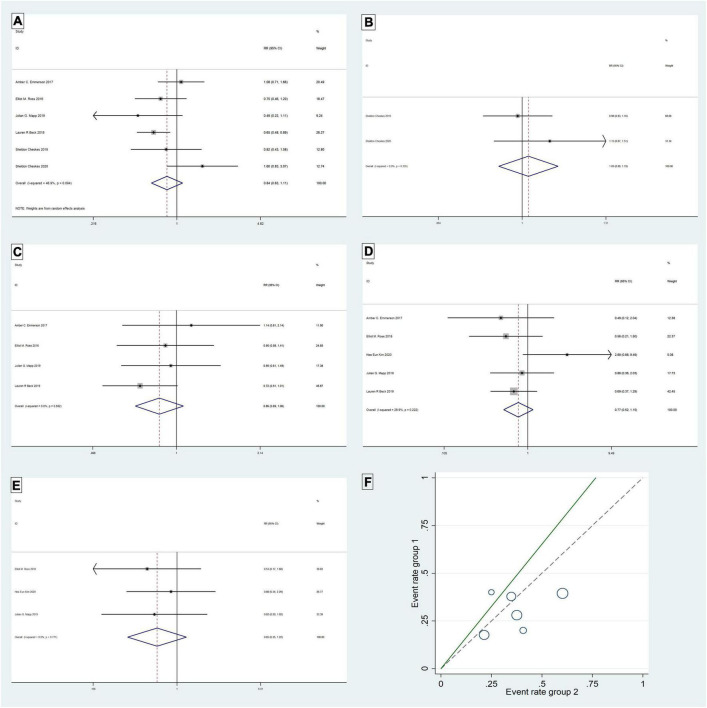
Return of spontaneous circulation: Forest plot **(A)**; L’ Abbe Plot **(F)**. Termination of refractory ventricular fibrillation: Frest plot **(B)**. Survival to hospital admission: Forest plot **(C)**. Survival to hospital discharge: Forest plot **(D)**. Good neurologic outcome: Forest plot **(E)**.

**FIGURE 3:**
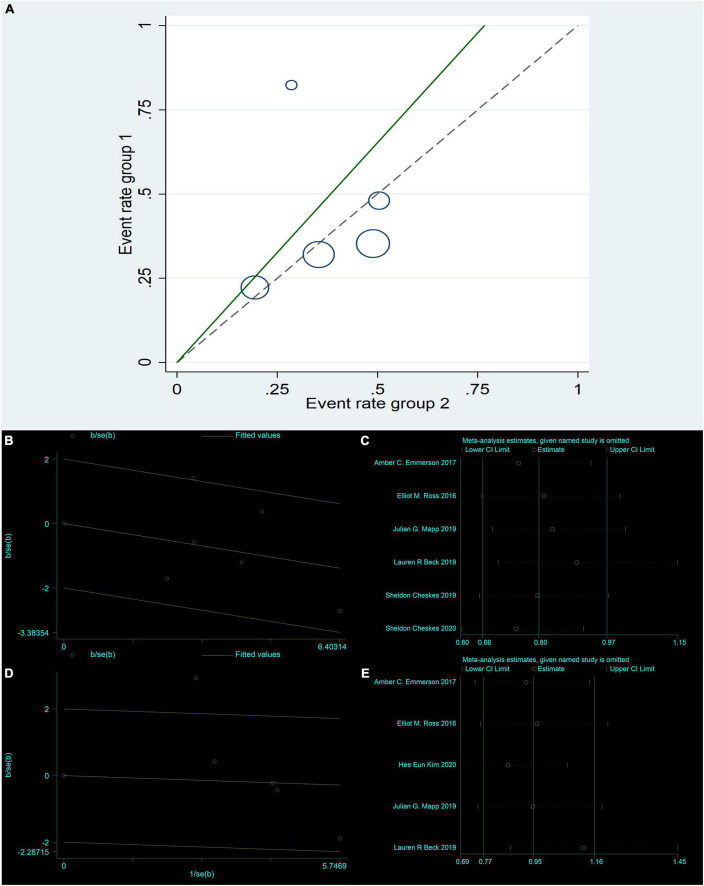
Return of spontaneous circulation: Radial Plot **(B)**; Sensitivity analysis **(C)**. Survival to hospital admission: L’Abbe Plot **(A)**; Radial Plot **(D)**; Sensitivity analysis **(E)**.

The six studies (4, 8, 14, 17, 19, 21) were tested for heterogeneity (*I^2^* = 46.9% < 50%), and *P* = 0.094 < 0.1 of the Q-test, suggesting that the heterogeneity among the article selected for this study was statistically significant, and further examining L’ Abbe Plot (Figure 2F) and Radial Plot (Figure 3B), there may be literature with high heterogeneity that requires a heterogeneity search. Sensitivity analysis of the current six studies (4, 8, 14, 17, 19, 21) revealed that removing any one study had little effect on the effect variables combined in the meta-analysis (Figure 3C). Although there was heterogeneity in the six studies (4, 8, 14, 17, 19, 21) (*I^2^* = 46.9% < 50%, *P* = 0.094 < 0.1), the heterogeneity was acceptable for the meta-analysis using a random-effects model. The RR value for the six studies (4, 8, 14, 17, 19, 21) pooled using random-effects model was 0.84 (95% CI, 0.63 to 1.11), which was not statistically significant (*Z* = 1.23, *P* = 0.219 > 0.05). The details are shown in the following forest plot (Figure 2A). The Begg’s Test resulted in *P* = 0.707 > 0.05, implying publication bias was not present in the six studies (4, 8, 14, 17, 19, 21).

The analysis of the study showed no difference between DSED and standard defibrillation in the return of spontaneous circulation.

#### Termination of refractory ventricular fibrillation

Two studies (4, 8) were tested for heterogeneity (*I^2^* = 0% < 50%) and *P* = 0.335 > 0.1 of the Q-test, suggesting that the heterogeneity between the literature selected for this study was not statistically significant and that the fixed-effects model could be selected for meta-analysis. The RR value for the two studies (4, 8) pooled using the fixed-effects model was 1.03 (95% CI, 0.89 to 1.19) and was not statistically significant (*Z* = 0.42, *P* = 0.678 > 0.05). The details are shown in the forest plot (Figure 2B). The Begg’s Test resulted in *P* = 1 > 0.05, implying that there is no publication bias in the two studies (4, 8).

There was no significant difference in the efficacy of DSED versus standard defibrillation in the termination of RVF.

#### Survival to hospital admission

Five studies (14, 17–19, 21) were tested for heterogeneity (*I^2^* = 67.5% > 50%) and *P* = 0.015 < 0.1 of the Q-test, suggesting that the heterogeneity among the selected studies for this study was statistically significant, and further examining the L’Abbe Plot (Figure 3A) and Radial Plot (Figure 3D), there was a strong possibility of heterogeneity in one study, and thus requiring the search for heterogeneity to be conducted. The sensitivity analysis of the five studies (14, 17–19, 21) revealed that Hee Eun Kim 2020 (18) had a large effect on heterogeneity, and after removing this study that the meta-analysis combined a large effect variable (Figure 3E). Therefore, the results of the heterogeneity test performed again after removing this study showed that heterogeneity existed in the remaining four studies (14, 17, 19, 21) (*I^2^* = 0% < 50%, *P* = 0.55 > 0.1), and the fixed-effects model was used for the meta-analysis. The RR value for the four studies (14, 17, 19, 21) using fixed-effects pooling was 0.86 (95% CI, 0.69 to 1.06), which was not statistically significant (*Z* = 1.4, *P* = 0.162 > 0.05). The details are shown in the forest plot (Figure 2C). The Begg’s Test resulted in *P* = 0.089 > 0.05, implying that there is no publication bias in the four studies (14, 17, 19, 21).

The DSED was not superior to standard defibrillation in the treatment of survival to hospital admission outcomes in patients with RVF, and there was no significant difference between the two comparisons.

#### Survival to hospital discharge

The meta-analysis of the five studies (14, 17–19, 21) using a fixed-effects model, after the heterogeneity test (*I^2^* = 29.9% < 50%) and *P* = 0.222 > 0.1 of the Q-test, suggested that the heterogeneity between the literature selected for this study was not statistically significant, five studies (14, 17–19, 21) were without heterogeneity, the RR value using fixed-effects pooling was 0.77 (95%CI, 0.52 to 1.15) and was not statistically significant (*Z* = 1.26, *P* = 0.206 > 0.05). The details are shown in the forest plot (Figure 2D). The Begg’s Test resulted in *P* = 1>0.05, implying that there is no publication bias in the five studies (14, 17–19, 21).

The use of DSED was not superior to standard defibrillation in the survival to hospital discharge of patients with RVF, and there was no significant difference between the two in the survival to hospital discharge outcome.

#### Good neurologic outcome (CPC 1∼2)

The meta-analysis of the three studies (18, 19, 21) using a fixed-effects model, after the heterogeneity test (*I^2^* = 0% < 50%) and *P* = 0.771 > 0.1 of the Q-test, suggested that the heterogeneity between the literature selected for this study was not statistically significant. Three studies (18, 19, 21) were without heterogeneity, the RR value using fixed-effects pooling was 0.65 (95%CI, 0.35 to 1.22) and was not statistically significant (*Z* = 1.33, *P* = 0.184 > 0.05). The details are shown in the forest plot (Figure 2E). The Begg’s Test resulted in *P* = 0.296 > 0.05, implying that there is no publication bias in the three studies (18, 19, 21).

Double sequential external defibrillation (DSED) was not superior to standard defibrillation in terms of survival to good neurological outcomes in patients with RVF, and there was no significant difference between the two in good neurological recovery outcomes.

### Secondary outcomes

#### Age

**FIGURE 4:**
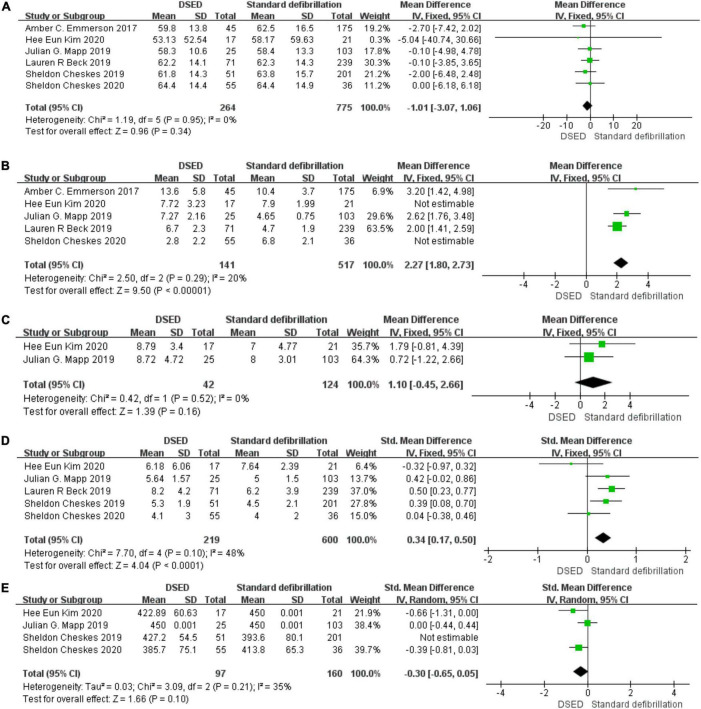
Age: Forest plot **(A)**. Total defibrillation attempts: Forest plot **(B)**. Emergency Medical System arrival time: Forest plot **(C)**. Epinephrine: Forest plot **(D)**. Amiodarone: Forest plot **(E)**.

Six studies (4, 8, 14, 17–19) were tested for heterogeneity (*I^2^* = 0% < 50%) and *P* = 0.95 > 0.1 of the Q-test, indicating that there was no heterogeneity among the selected studies and that the fixed-effects model could be selected for meta-analysis. Sensitivity analysis was continued to ensure the accuracy and stability of the study. Sensitivity analysis was performed on these six studies (4, 8, 14, 17–19), and none of them significantly interfered with the results of this meta-analysis, implying that this study has good stability. the MD value of the six studies (4, 8, 14, 17–19) using fixed-effects pooling was −1.01 (95%, −3.07 to 1.06), which was not statistically significant (*Z* = 0.96, *P* = 0.34 > 0.05). The details are shown in the forest plot (Figure 4A).

In comparing the age of patients with RVF, there was no statistically significant difference between the two groups using DSED and standard defibrillation. Therefore, the age factor did not have a significant effect on the outcome.

#### Total defibrillation attempts

Five studies (4, 14, 17–19) were tested for heterogeneity (*I^2^* = 97% > 50%) and *P* = 0.001 < 0.1 of the Q-test, suggesting heterogeneity among the papers selected for this study. The sensitivity analysis of five studies (4, 14, 17–19) revealed that Hee Eun Kim 2020 (18) and Sheldon Cheskes 2020 (4) studies caused great interference with the results of this meta-analysis and after the removal of two studies (*I^2^* = 20% < 50%), and *P* < 0.001 of the Q-test, there was no heterogeneity the studies selected for the fixed-effects model for meta-analysis. The MD value for the three studies (14, 17, 19) pooled using fixed-effects model was 2.27 (95%, 1.80 to 2.73) with statistical significance (*Z* = 9.50, *P* = 0.001 < 0.05). The details are shown in the forest plot (Figure 4B).

There was a difference in the number of defibrillations between DSED and standard defibrillation when treating patients with RVF. The number of defibrillations during the rescue process may have a significant impact on the outcome.

#### Emergency medical system arrival time

Two studies (18, 19) were tested for heterogeneity (*I^2^* = 0% < 50%) and *P* = 0.52 > 0.1 of the Q-test, suggesting that there was no heterogeneity between the studies selected for this study and that the fixed-effects model could be selected for meta-analysis. Sensitivity analysis was continued to ensure the accuracy and stability of the study. Sensitivity analysis was performed on the two studies (18, 19), and none of the studies caused much interference with the results of this meta-analysis, implying that this study has good stability. The MD value for the two studies (18, 19) using fixed-effects pooling was 1.10 (95%, −0.45 to 66), which was not statistically significant, (*Z* = 1.39, *P* = 0.16 > 0.05). The details are shown in the forest plot (Figure 4C).

Comparing the DSED and standard defibrillation groups during the rescue of RVF patients was not statistically significant, There was no significant difference in the emergency medical system arrival time between the two groups.

#### Epinephrine

Five studies (4, 8, 14, 18, 19) were tested for heterogeneity (*I^2^* = 48% < 50%) and *P* = 0.1 of the Q-test, suggesting that the mild heterogeneity between the studies selected for this study was within acceptable limits, and fixed-effects model was selected for meta-analysis. Sensitivity analysis was continued to ensure the accuracy and stability of the study. Sensitivity analysis was performed on these five studies (4, 8, 14, 18, 19), and none of them significantly interfered with the results of this meta-analysis, implying that this study has good stability. The SMD value for the fixed-effects summary of the five studies (4, 8, 14, 18, 19) was 0.34 (95%, 0.17 to 0.50) with statistically significant (*Z* = 4.04, *P* = 0.001 < 0.05). The details are shown in the following forest plot (Figure 4D).

The epinephrine dose in patients on DSED was 0.34 times higher than standard defibrillation, with a statistically significant difference. The epinephrine dose during rescue may have a more significant effect on the outcome.

#### Amiodarone

Four studies (4, 8, 18, 19) were tested for heterogeneity (*I*^2^ = 80% > 50%) and *P* = 0.002 < 0.1 of the Q-test, suggesting heterogeneity among the studies selected for this study. Sensitivity analysis of the four papers in this study revealed that the Sheldon Cheskes 2019 study (8) caused significant interference with the results of this meta-analysis, so the results of the heterogeneity test conducted again after excluding this study showed that there was no heterogeneity in the remaining three studies (4, 18, 19) (*I^2^* = 35% < 50%, *P* = 0.21 > 0.1), and a random-effects model was used for the meta-analysis. The SMD value for the three studies (4, 18, 19) pooled using random-effects was -0.30 (95%, -0.65 to-0.05), which was not statistically significant (*Z* = 1.66, *P* = 0.1 > 0.05). The details are shown in the following forest plot (Figure 4E).

There was no difference in the dose of amiodarone used in patients during the rescue with DSED versus standard defibrillation, which was not statistically significant. There were no significant differences in the use of amiodarone doses during the rescue.

### Quality of the evidence

From the nine included observational studies (8, 14–21), we assessed the overall risk of bias as critical in six studies and serious in three studies (Table 2). The critical risk of bias was mainly a result of a critical risk of confounding due to a lack of adjusting for covariates. There were also studies with an increased risk of bias and missing data due to patient selection. The risk of bias in one pilot RCT study (4) was assessed as having some problems with the risk of bias and clinical heterogeneity among them. The quality of the studies of the 10 studies (4, 8, 14–21) included remains to be improved.

**TABLE 2:** Risk of bias of included studies.

Author	Confounding	Selection	Classification of interventions	Deviation from intended intervention	Missing data	Outcomes	Selective reporting	Overall
**Observational studies using Robins-I**								
Emmerson et al. (17)	Critical	Low	Low	Moderate	Low	Low	Low	Critical
Cheskes et al. (8)	Critical	Moderate	Low	Moderate	Moderate	Low	Low	Critical
Merlin et al. (20)	Critical	Serious	Low	Low	Low	Low	Low	Critical
Cabañas et al. (15)	Critical	Serious	Low	Low	Critical	Low	Low	Critical
Ross et al. (21)	Critical	Low	Low	Moderate	Moderate	Low	Low	Critical
Beck et al. (14)	Serious	Low	Low	Low	Low	Low	Low	Serious
Mapp et al. (19)	Serious	Moderate	Moderate	Low	Serious	Low	Low	Serious
Kim et al. (18)	Serious	Low	Low	Low	Low	Low	Low	Serious
Cortez et al. (16)	Critical	Critical	Low	Moderate	Low	Low	Low	Critical

**Author**	**Randomization**	**Selection**	**Deviation from intended intervention**	**Missing outcome**		**Outcome measurement**	**Selective reporting**	**Overall**

**Randomized controlled trial using ROB-2**							
Cheskes et al. (4)	Some concern	Some concern	Some concern	Low		Low	Low	Some concern

## Discussion

This systematic review and meta-analysis summarize 10 studies (4, 8, 14–21) that looked at the impact of DSED on primary outcomes and associated factors affecting outcomes in comparison with standard defibrillation in the treatment of refractory ventricular fibrillation. A 2019 article (22) shows that no evidence was found that DSED was associated with improved survival outcomes in patients with RVF. Survival to hospital discharge, event survival, and ROSC were analyzed in only two studies. And there was confounding bias in the two included studies. A similar study was conducted in an article in 2020 (7). However, the emergency medical system arrival time, the critical defibrillation frequency, and the use of epinephrine and amiodarone were not considered in these two studies (7, 22). The DSED has been studied for decades in the electrophysiology lab for patients in RVF (8). The exact mechanism by which patients respond to DSED remains unknown. Animal studies have suggested that DSED may reduce the defibrillation threshold (23). Transthoracic impedance has also been found to be decreased by sequential defibrillations, resulting in higher current density at the cardiac surface (8, 24). Some studies have shown that DSED has a superior prognosis to standard defibrillation in the early stages, while others have shown the opposite (8, 14). The main reason for this may be clinical heterogeneity leading to inconsistent use of DSED in clinical practice. Although there are studies showing that DSED may be successful in the treatment of RVF, a well-designed, high-quality, multicenter randomized trial is needed to elucidate the efficacy and role of DSED in the treatment of refractory VF. In the recent Sheldon Cheskes study (25) of patients with refractory ventricular fibrillation, it was shown that patients who received DSED defibrillation were more likely to survive to hospital discharge than those who received standard defibrillation. The trial was terminated early because of operational challenges. The trial did not achieve the planned sample size and did not provide information about post-resuscitation care It is possible that the treatment effect was overestimated, given the small number of events for the primary outcome. However, additional studies are needed to further determine the effect of DSED defibrillation on refractory ventricular fibrillation, including adequate sample sizes and incorporating details related to postresuscitation care.

A total of 1347 patients were enrolled in this study to assess whether DSED is more advantageous in the treatment of refractory ventricular fibrillation by observing the primary outcome. Heterogeneity tests, sensitivity analyses by L’ Abbe Plot and Radial Plot, forest plot representation by fixed-effects model or random-effects model, and bias tests by Begg’s test were used to draw the conclusions. The DSED was found to have no advantage in comparison with standard defibrillation for refractory ventricular fibrillation for ROSC, termination of RVF, survival to admission, survival to discharge, and good neurological outcome, with no statistically significant difference in the comparison.

The analysis of the relevant factors affecting the outcome revealed differences and statistical significance in the comparison of the number of defibrillations and the dose of epinephrine used. There were no statistically significant differences in age, emergency medical system arrival time, or amiodarone dose, suggesting better homogeneity between the two groups in terms of these factors. The DSED did not show a significant advantage over standard defibrillation in the primary outcome, possibly because the number of defibrillations and epinephrine dose used had an impact on the outcome indicators, making the two groups less homogeneous and thus affecting the outcome. In this study, the number of defibrillations and the dose of epinephrine may have an impact on the results. The DSED was performed after three shocks, and the paramedics applied a second set of defibrillation pads and used two defibrillators to deliver two almost simultaneous defibrillation shocks for defibrillation attempts. Therefore, the total number of defibrillations was higher in the DSED group. Physiologic increases in the plasma epinephrine concentration may increase the number and energy of shocks needed to terminate VF (26), the DSED group used higher doses of epinephrine than the standard group and had more defibrillations, which suggests that the primary outcome may be altered by changing the number of defibrillations and epinephrine dose. There are still few studies on the relationship between epinephrine and the number of defibrillations, and the evidence is insufficient. Therefore, further clinical studies are needed.

### Limitations and strengths

Our study has limitations. First, to safeguard the population in which the intervention was implemented in the study, we excluded various effects of passersby on the study that could have had additional effects on outcome indicators. Second, the literature search was limited to publications that were in English, and most of the studies we included were observational and susceptible to receiving confounding factors, as we attempted to reduce these effects by assessing and reporting the risk of bias. Third, with only 1 study in the entire study being an RCT and a limited sample size, the study still needs a large multicenter randomized controlled study to ensure the rigor of the study. Despite these limitations, our systematic review also has several strengths. This is the first analysis that combines the effects of relevant influencing factors on outcome indicators, ensuring the rigor of the systematic review and meta-analysis, controlling for confounding factors in the study, improving the quality of the study, and making it more convincing.

## Conclusion

This systematic review and meta-analysis compared the effect of DSED and standard defibrillation on the outcome of rescue in patients with RVF and did not find differences in termination of RVF, ROSC, survival to hospital admission, survival to hospital discharge, good neurological outcome, emergency medical system arrival time, and amiodarone doses. However, some differences were found in the number of defibrillations and the dose of epinephrine used during rescue. Considering the high number of confounding factors in this type of study, there is still a need to work on expanded clinical trials for this population and use real-world research data.

## Data availability statement

The original contributions presented in this study are included in the article/supplementary material, further inquiries can be directed to the corresponding author.

## Author contributions

YL designed the research question and drafted the manuscript. YL and JY reviewed all abstracts, selected full text for inclusion, and performed data abstraction. DL adjudicated any disagreements. XH, ZL, and XY performed the bulk of the analysis with input from YL. JY takes responsibility for the manuscript as a whole. All authors contributed substantially to its revision.
